# Human Betacoronavirus 2c EMC/2012–related Viruses in Bats, Ghana and Europe

**DOI:** 10.3201/eid1903.121503

**Published:** 2013-03

**Authors:** Augustina Annan, Heather J. Baldwin, Victor Max Corman, Stefan M. Klose, Michael Owusu, Evans Ewald Nkrumah, Ebenezer Kofi Badu, Priscilla Anti, Olivia Agbenyega, Benjamin Meyer, Samuel Oppong, Yaw Adu Sarkodie, Elisabeth K.V. Kalko, Peter H.C. Lina, Elena V. Godlevska, Chantal Reusken, Antje Seebens, Florian Gloza-Rausch, Peter Vallo, Marco Tschapka, Christian Drosten, Jan Felix Drexler

**Affiliations:** Author affiliations: Kumasi Centre for Collaborative Research in Tropical Medicine, Kumasi, Ghana (A. Annan, M. Owusu);; Macquarie University, Sydney, New South Wales, Australia (H.J. Baldwin);; University of Ulm, Ulm, Germany (H.J. Baldwin, S.M. Klose, E.K.V. Kalko, P. Vallo, M. Tschapka);; University of Bonn Medical Centre, Bonn, Germany (V.M. Corman, B. Meyer, F. Gloza-Rausch, C. Drosten, J.F. Drexler);; Kwame Nkrumah University of Science and Technology, Kumasi (E.E. Nkrumah, E.K. Badu, P. Anti, O. Agbenyega, S. Oppong, Y.A. Sarkodie);; Smithsonian Tropical Research Institute, Balboa, Panama (E.K.V. Kalko, M. Tschapka);; Naturalis Biodiversity Center, Leiden, the Netherlands (P.H.C. Lina);; Schmalhausen Institute of Zoology, Kiev, Ukraine (E.V. Godlevska);; Netherlands Center for Infectious Disease Control, Bilthoven, the Netherlands (C. Reusken); Noctalis, Centre for Bat Protection and Information, Bad Segeberg, Germany (A. Seebens, F. Gloza-Rausch);; Academy of Sciences of the Czech Republic, v.v.i., Brno, Czech Republic (P. Vallo)

**Keywords:** Africa, Europe, Ghana, coronavirus, bats, human betacoronavirus, CoV-EMC, viruses

## Abstract

We screened fecal specimens of 4,758 bats from Ghana and 272 bats from 4 European countries for betacoronaviruses. Viruses related to the novel human betacoronavirus EMC/2012 were detected in 46 (24.9%) of 185 *Nycteris* bats and 40 (14.7%) of 272 *Pipistrellus* bats. Their genetic relatedness indicated EMC/2012 originated from bats.

Coronaviruses (CoVs) are enveloped viruses with a positive-sense, single-stranded RNA genome (*1*). CoVs are classified into 4 genera: *Alphacoronavirus*, *Betacoronavirus* (grouped further into clades 2a–2d), *Gammacoronavirus*, and *Deltacoronavirus*. Two human coronaviruses (hCoVs), termed hCoV-OC43 and −229E, have been known since the 1960s and cause chiefly mild respiratory disease (*2*). In 2002–2003, an outbreak of severe acute respiratory syndrome (SARS) leading to ≈850 deaths was caused by a novel group 2b betacoronavirus, SARS-CoV (*3*). A likely animal reservoir for SARS-CoV was identified in rhinolophid bats (*4*,*5*). In the aftermath of the SARS pandemic, 2 hCoVs, termed hCoV-NL63 and -HKU1, and numerous novel bat CoVs were described.

In September 2012, health authorities worldwide were notified of 2 cases of severe respiratory disease caused by a novel hCoV (*6*,*7*). This virus, termed EMC/2012, was related to the 2c betacoronavirus clade, which had only been known to contain *Tylonycteris bat coronavirus* HKU4 and *Pipistrellus bat coronavirus* HKU5 (*8*).

We previously identified highly diversified alphacoronaviruses and betacoronaviruses, but not clade 2c betacoronaviruses, in bats from Ghana (*9*). We also identified sequence fragments from a 2c betacoronavirus from 1 *Pipistrellus* bat in Europe (*10*). In this study, we analyzed an extended sample of 4,758 bats from Ghana and 272 bats from 4 European countries.

## The Study

**Figure 1:**
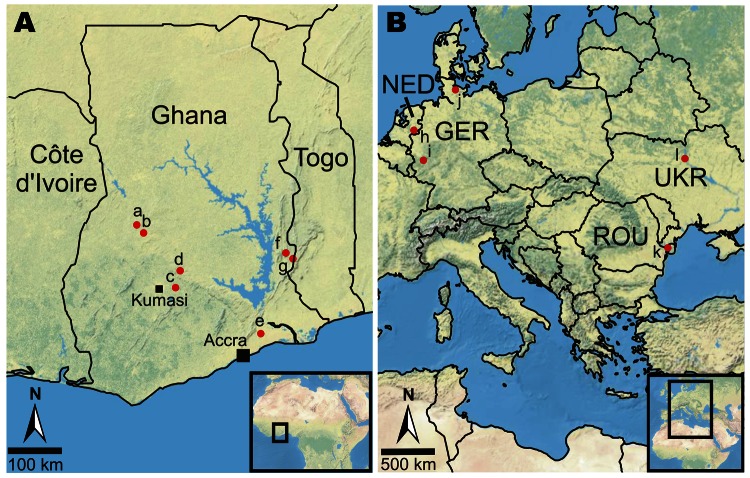
Location of bat sampling sites in Ghana and Europe. The 7 sites in Ghana (A) and the 5 areas in Europe (B) are marked with dots and numbered from west to east. a, Bouyem (N7°43′24.899′′ W1°59′16.501′′); b, Forikrom (N7°35′23.1′′ W1°52′30.299′′); c, Bobiri (N6°41′13.56′′ W1°20′38.94′′); d, Kwamang (N6°58′0.001′′ W1°16′0.001′′); e, Shai Hills (N5°55′44.4′′ E0°4′30′′); f, Akpafu Todzi (N7°15′43.099′′ E0°29′29.501′′); g, Likpe Todome (N7°9′50.198′′ E0°36′28.501′′); h, Province Gelderland, NED (N52°1′46.859′′ E6°13′4.908′′); i, Eifel area, federal state Rhineland-Palatinate, GER (N50°20′5.316′′ E7°14′30.912′′); j, Holstein area, federal state Schleswig-Holstein, GER (N54°14′51.271′′ E10°4′3.347′′); k, Tulcea county, ROU (N45°12′0.00′′ E29°0′0.00′′); l, Kiev region, UKR (N50°27′0.324′′ E30°31′24.24′′). NED, the Netherlands; GER, Germany; ROU, Romania; UKR, Ukraine.

Fecal specimens were collected from 10 bat species in Ghana and 4 *Pipistrellus* species in Europe (Table 1). Bats were caught during 2009–2011 with mist nets, as described (*9*), in 7 locations across Ghana and 5 areas in Germany, the Netherlands, Romania, and Ukraine (Figure 1). The species, age, sex, reproductive status, and morphologic measurements of the bats were recorded. Fecal pellets were collected and suspended in RNAlater Stabilization Reagent (QIAGEN, Hilden, Germany). RNA was purified as described (*11*). CoV was detected by using nested reverse transcription PCR (RT-PCR) targeting the *RNA-dependent RNA polymerase* (*RdRp*) gene (*12*) (see Table 1 for assay oligonucleotides).

**Table 1:** Overview of bats tested for 2c betacoronaviruses, Ghana and Europe

Area, bat species	No. bats tested (no. [%] positive)*	Age, juvenile/adult†	Sex, F/M‡	Location§ (no. tested/no. positive)
Ghana				
* Coleura afra*	108 (0)	2/105	46/59	a, b, e
* Hipposideros abae*	604 (0)	55/548	207/341	a, b, d, f
* H. cf. gigas*	28 (0)	7/19	8/11	a, b, d
* H. fuliginosus*	1 (0)	1/0	Unknown	c
* H. jonesi*	31 (0)	6/25	1/24	c, d
* H. cf. ruber*	3,763 (0)	674/3,078	1,109/1,969	a, b, c, d, f, g
* Nycteris cf. gambiensis*	185 (46 [24.9])	22/161¶	79/82	a# (5/2), b# (65/15), d# (104/29), f (1/0)
* Rhinolophus alcyone*	4 (0)	2/2	1/1	c
* R. landeri*	13 (0)	3/10	2/8	b, d, f
* Taphozous perforatus*	21 (0)	3/18	0/18	e
Total	4,758 (46 [1.0])			
Europe				
* Pipistrellus kuhlii*	7 (0)	Unknown	3/3	l
* P. nathusii*	82 (30 [36.6])	15/65	38/43	j (2/0), k# (74/29), l# (6/1)
* P. pipistrellus*	42 (1 [2.4])	17/25	19/21	i (29/0), k# (7/1), h (6/0)
* P. pygmaeus*	141 (9 [6.4])	11/127	83/55	j (44/0), k# (91/9), l (6/0)
Total	272 (40 [14.7])			

*The real-time reverse transcription PCR (Ghana) used oligonucleotides 2c-rtF, 5′-GCACTGTTGCTGGTGTCTCTATTCT-3′, 2crtR, 5′-GCCTCTAGTGGCAGCCATACTT-3′ and 2c-rtP, JOE-TGACAAATCGCCAATACCATCAAAAGATGC-BHQ1 and the Pan2c-heminested assay (Europe) used oligonucleotides Pan2cRdRP-R, 5′-GCATWGCNCWGTCACACTTAGG-3′; Pan2cRdRP-Rnest, 5′-CACTTAGGRTARTCCCAWCCCA-3′; and Pan2cRdRp-FWD, 5′-TGCTATWAGTGCTAAGAATAGRGC-3′. †Excludes bats (all coronavirus-negative) that were missing data for age. ‡ Excludes bats that were missing data for sex. §a, Bouyem; b, Forikrom; c, Bobiri; d, Kwamang; e, Shai Hills; f, Akpafu Todzi, g, Likpe Todome; h, Province Gelderland; i, Eifel area; j, Holstein area; k,Tulcea county; l, Kiev region; GPS coordinates are shown in Figure 1. ¶For 2 animals, no data on age were available. #Locations in which coronavirus 2c–positive bats were found.

A novel CoV was detected in insectivorous *Nycteris cf. gambiensis* specimens (online Technical Appendix Figure, wwwnc.cdc.gov/EID/pdfs/12-1503-Techapp.pdf; GenBank accession nos. JX899382–JX899384). A real-time RT-PCR was designed to permit sensitive and quantitative detection of this CoV (Table 1**)**. Only *Nycteris* bats were positive for CoV (46 [24.9%] of 185 specimens) (Table 1). Demographic factors predictive of CoV in captured *Nycteris* bats were assessed. Juvenile bats and lactating females were significantly more likely to be CoV-infected than were adult and nonlactating female bats, respectively (Table 2). Virus concentrations in feces from *Nycteris* bats were high (median 412,951 RNA copies/g range 323–150,000,000 copies/g).

**Table 2:** Possible factors predictive of 2c betacoronavirus detection in *Nycteris cf. gambiensis* bats, Ghana and Europe*

Variable	No. tested	CoV positive, no. (%)	χ^2^	p value	Odds ratio (95% CI)
Age					
Juvenile	22	10 (45.4)	5.49	0.02	2.89 (1.16–7.24)
Adult	161	36 (22.4)			
Sex					
F	79	16 (20.3)	0.01	0.91	1.04 (0.50–2.17)
M	82	20 (24.4)			
Lactation status, F					
Lactating	25	11 (44.0)	12.77	0.0004	7.70 (2.29–25.89)
Nonlactating	54	5 (9.3)			
Gravidity, F					
Gravid	13	0	3.95	0.06†	0
Nongravid	66	16 (24.2)			
Reproductive status, M					
Active	56	15 (26.8)	0.55	0.46	1.54 (0.49–4.81)
Nonreproductive	26	5 (19.2)			

*All analyses, except for the gravity parameter (because 1 of the expected values was <5), were done by using uncorrected χ^2^ tests (2-tailed) in Epi Info 7 (wwwn.cdc.gov/epiinfo/7). All analyses except age excluded juvenile bats. †Fisher exact test.

The 398-bp CoV *RdRp* screening fragment was extended to 816 bp, as described (*5*), to enable more reliable taxonomic classification. We previously established *RdRp*-grouping units (RGU) as a taxonomic surrogate to enable prediction of CoV species on the basis of this 816-bp fragment when no full genome sequences could be obtained. According to our classification, the amino acid sequences in the translated 816-bp fragment of the tentative betacoronavirus species (RGU) differed from each other by at least 6.3% (*5*). The new *Nycteris* bat CoV differed from the 2c-prototype viruses HKU4 and HKU5 by 8.8%–9.6% and from EMC/2012 by 7.5% and thus constituted a novel RGU. A partial *RdRp* sequence fragment of a *P. pipistrellus* bat CoV from the Netherlands, termed VM314 (described by us in [*10*]), was completed toward the 816-bp fragment to refine the RGU classification of EMC/2012. EMC/2012 differed from VM314 by only 1.8%.

Because of the genetic similarity between EMC/2012 and VM314, we specifically investigated *Pipistrellus* bats from 4 European countries for 2c betacoronaviruses. We detected betacoronaviruses in 40 (14.7%) of 272 *P. pipistrellus*, *P. nathusii*, and *P. pygmaeus* bats from the Netherlands, Romania, and Ukraine (Table 1; GenBank accession nos. KC243390-KC243392) that were closely related to VM314. The VM314-associated *Pipistrellus* bat betacoronaviruses differed from EMC/2012 by 1.8%. The difference between EMC/2012 and HKU5 was 5.5%–5.9%. In summary, HKU5, EMC/2012, and the VM314-associated clade form 1 RGU according to our classification system, and the VM314-*Pipistrellus* bat clade contains the closest relatives of EMC/2012. HKU4 and the *Nycteris* CoV define 2 separate tentative species in close equidistant relationship.

**Figure 2:**
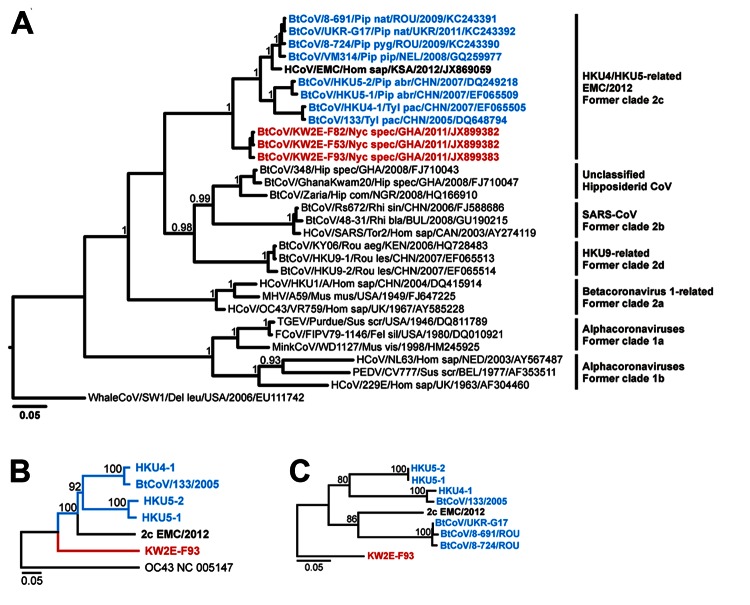
*RNA-dependent RNA polymerase* (*RdRp*) gene and *Spike* genephylogenies including the novel betacoronaviruses from bats in Ghana and Europe. A) Bayesian phylogeny of an 816-nt *RdRp* gene sequence fragment corresponding to positions 14781–15596 in severe acute respiratory syndrome coronavirus (SARS-CoV) strain Frankfurt 1 (GenBank accession no. AY291315). Data were analyzed with MrBayes version 3.1 (http://mrbayes.sourceforge.net/) by using a WAG amino acid substitution model and 4 million generations sampled every 100 steps. Trees were annotated by using a burn-in of 10,000 and visualized with FigTree version 1.6.1 from the BEAST package (www.beast.bio.ed.ac.uk). A whale gammacoronavirus was used as an outgroup. The novel *Nycteris* bat viruses are shown in **boldface** and red, the novel *Pipistrellus* bat viruses and other bat CoVs in the 2c clade are shown in **boldface** and cyan, and the novel human betacoronavirus EMC/2012 is shown in **boldface**. Values at deep nodes represent statistical support of grouping by posterior probabilities. CoV clades are depicted to the right of taxa. B) Phylogeny of the complete *Spike* gene of clade 2c CoVs determined by using the neighbor-joining method with an amino acid percentage distance substitution model and the complete deletion option in MEGA5 (www.megasoftware.net). The Nycteris CoV *Spike* gene was equidistant from other 2c-CoV *Spike* genes with 45.6%–46.8% aa divergence. Human coronavirus (hCoV)–OC43 was used as an outgroup. No complete *Spike* gene sequence was available for VM314 or the novel *Pipistrellus* bat CoVs. Scale bar represents percentage amino acid distance. The analysis comprised 1,731 aa residues. C) Phylogeny of the partial *Spike* gene of clade 2c CoVs, including the novel CoVs of *Pipistrellus* bats from Europe, determined by using a nucleotide distance substitution model and the complete deletion option in MEGA5. Scale bar represents percentage nucleotide distance. The analysis comprised 131 nt corresponding to positions 25378–25517 in hCoV-EMC/2012. Oligonucleotide sequences of primers used to amplify full and partial *Spike* gene sequences are available on request from the authors. Values at deep nodes in B and C represent statistical support of grouping by percentage of 1,000 bootstrap replicates. GenBank accession numbers for the complete and partial *Spike* genes correspond to those given in panel A for the *RdRp* gene.

We conducted a Bayesian phylogenetic analysis. In this analysis, the *Nycteris* bat CoV clustered as a phylogenetically basal sister clade with HKU4, HKU5, and EMC/2012 and the associated European *Pipistrellus* viruses (Figure 2).

To confirm the *RdRp*-based classification, we amplified the complete glycoprotein-encoding *Spike* gene and sequenced it for the novel *Nycteris* bat virus. The phylogenetically basal position of the novel *Nycteris* bat virus within the 2c clade resembled that in the CoV *RdRp* gene (Figure 2). Partial sequences that could be obtained from the 3′-end of the *Spike* gene of three 2c *Pipistrellus* bat betacoronaviruses confirmed their relatedness to EMC/2012 (Figure 2).

## Conclusions

We detected novel clade 2c betacoronaviruses in *Nycteris* bats in Ghana and *Pipistrellus* bats in Europe that are phylogenetically related to the novel hCoV EMC/2012. All previously known 2c bat CoVs originated from vespertilionid bats: VM314 originated from a *P. pipistrellus* bat from the Netherlands and HKU4 and HKU5 originated from *Tylonycteris pachypus* and *P. abramus* bats, respectively, from the People’s Republic of China. The *Nycteris* bat virus in Africa extends this bat CoV clade over 2 different host families, Nycteridae and Vespertilionidae (Technical Appendix Figure). Detection of genetically related betacoronaviruses in bats from Africa and Eurasia parallels detection of SARS-CoV in rhinolophid bats from Eurasia and related betacoronaviruses in hipposiderid bats from Africa (*9*).

The relatedness of EMC/2012 to CoVs hosted by *Pipistrellus* bats at high prevalence across different European countries and the occurrence of HKU5 in bats of this genus from China highlight the possibility that *Pipistrellus* bats might indeed host close relatives of EMC/2012. This suspicion is supported by observations that tentative bat CoV species (RGUs) are commonly detected within 1 host genus (*5*). Within the Arabian Peninsula, the International Union for Conservation of Nature (www.iucn.org) lists 50 bat species, including *P. arabicus*, *P. ariel*, *P*. *kuhlii*, *P. pipistrellus*, *P. rueppellii*, and *P. savii* bats. Because of the epidemiologic link of EMC/2012 with the Arabian Peninsula (*6*,*7*), bats from this area should be specifically screened.

The genomic data suggest that EMC/2012, like hCoV-229E and SARS-CoV, might be another human CoV for which an animal reservoir of closely related viruses could exist in Old World insectivorous bats (*4*,*9*). Whether cross-order (e.g., chiropteran, carnivore, primate) host switches, such as suspected for SARS-CoV, have occurred for 2c clade bat CoVs remains unknown. However, we showed previously that CoVs are massively amplified in bat maternity colonies in temperate climates (*13*). This amplification also might apply to the *Nycteris* bat CoV because, as shown previously for vespertilionid bats from temperate climates (*14*), detection rates of CoV are significantly higher among juvenile and lactating *Nycteris* bats. In light of the observed high virus concentrations, the use of water from bat caves and bat guano as fertilizer for farming and the hunting of bats as wild game throughout Africa (*15*) may facilitate host switching events. To our knowledge, no CoV has been isolated directly from bats. Further studies should still include isolation attempts to obtain full virus genomes and to identify virulence factors that may contribute to the high pathogenicity of EMC/2012 (*7*).

Technical Appendix Figure Bat evolutionary lineages and species in which novel group 2c betacoronaviruses were detected, Ghana and Europe.
