# Supplementary material for: Human Betacoronavirus 2c EMC/2012–related Viruses in Bats, Ghana and Europe
Source: Emerg Infect Dis. 2013 Mar;19(3):456–9. doi: 10.3201/eid1903.121503 (PMC3647674; doi:10.3201/eid1903.121503)
Supplement: Technical Appendix Figure — Bat evolutionary lineages and species in which novel group 2c betacoronaviruses were detected, Ghana and Europe. [file 12-1503-Techapp-s1.pdf]

Article DOI: http://dx.doi.org/10.3201/eid1903.121503

Human Betacoronavirus 2c EMC/2012–related Viruses in Bats, Ghana and Europe

Technical Appendix

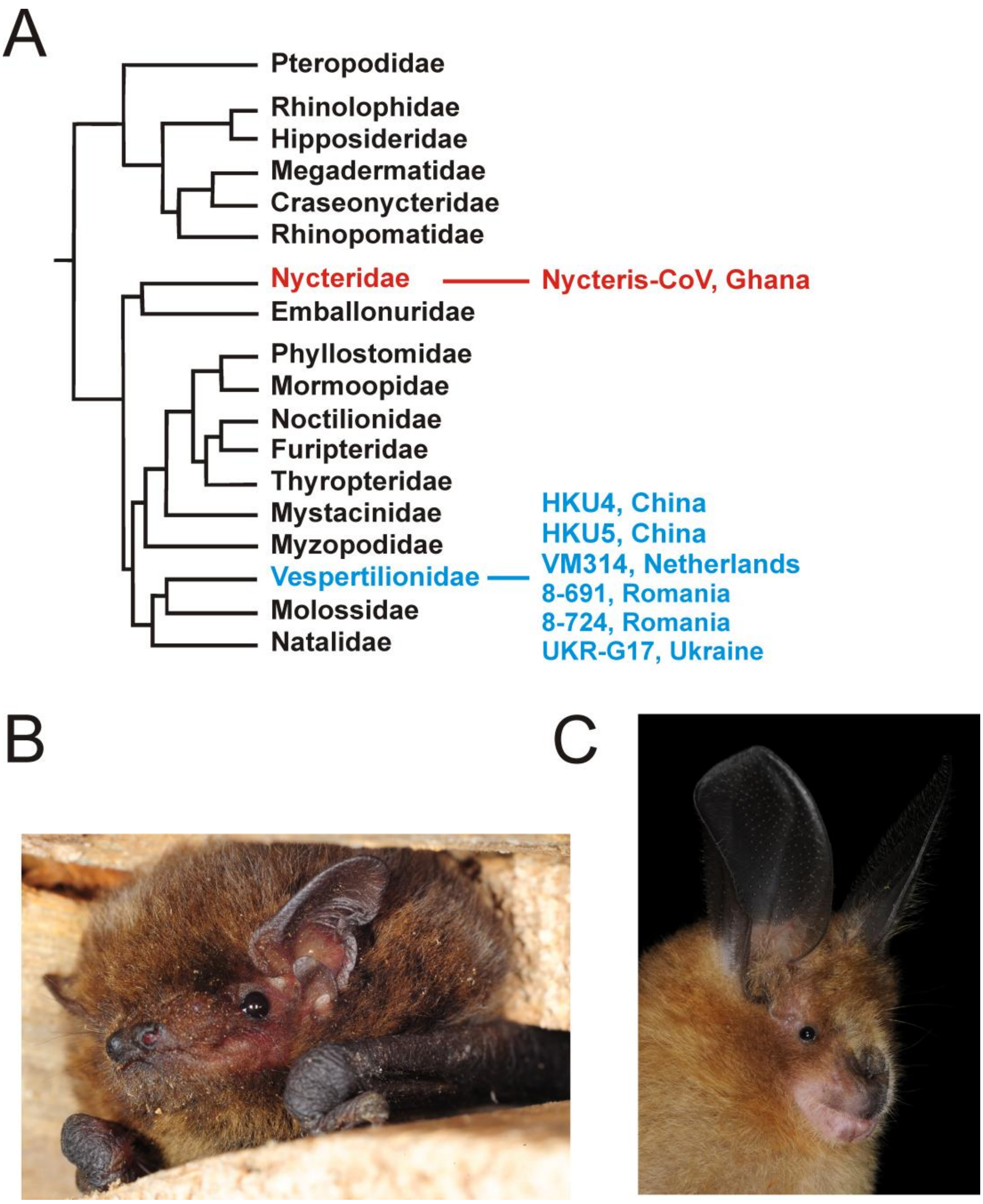

Technical Appendix Figure. Bat evolutionary lineages and species in which novel group 2c betacoronaviruses were detected, Ghana and Europe. A) Bat evolutionary lineages in which novel

group 2c betacoronaviruses were detected. Phylogeny adapted from (*1*). Bat families Nycteridae and Vespertilionidae and coronaviruses hosted by bats of these families detected in this and previous studies are shown in red and cyan, respectively. CoV, coronaviurs. B) European *Pipistrellus nathusii* bat; photo by Florian Gloza-Rausch. C) Ghanaian *Nycteris cf. gambiensis* bat; photo by Marco Tschapka.

Reference

1. Simmons NB. Evolution. An eocene big bang for bats. Science. 2005;307:527–8. PubMed http://dx.doi.org/10.1126/science.1108871
